# CD200fc enhances anti-tumoral immune response and inhibits visceral metastasis of breast carcinoma

**DOI:** 10.18632/oncotarget.24931

**Published:** 2018-04-10

**Authors:** Nuray Erin, Gamze Tanrıöver, Anna Curry, Muhlis Akman, Özlem Duymuş, Reg Gorczynski

**Affiliations:** ^1^ Department of Medical Pharmacology, Akdeniz University, School of Medicine, Antalya, Turkey; ^2^ Histology and Embryology, Akdeniz University, School of Medicine, Antalya, Turkey; ^3^ University Health Network, Toronto General Hospital, Toronto, Canada

**Keywords:** CD200fc, lung metastasis, liver metastasis, breast cancer

## Abstract

CD200 is a widely expressed cell surface glycoprotein that inhibits excessive inflammation in autoimmunity, transplantation, and viral infections. We previously observed that visceral metastasis of highly aggressive and inflammatory 4THM breast carcinoma cells was markedly decreased in CD200 transgenic mice. The goal of this study was to determine whether exogenous exposure to CD200fc mimics the effects of endogenously over expressed CD200. Female BALB/c mice were injected with CD200fc two times a week for five times. Injection was started two days after orthotopic injection of 4THM cells. Tumor infiltrating Gr1+Cd11b+ cells were decreased while CD8+ cells were increased in CD200fc-treated animals. CD200fc injection significantly decreased lung and liver metastasis and the growth of primary tumors. CD200fc injection enhanced the tumor-induced IFN-g response while suppressing the IL-10 response. We observed excessive basal IL-6 secretion in MLC which was significantly decreased in CD200fc treated mice 12 days after injection of 4TM cells. These results are in accord with previous data from CD200 transgenic mice, and demonstrate for the first time that CD200 analogues might have therapeutic potential in the treatment of aggressive breast carcinoma which induces excessive systemic inflammation.

## INTRODUCTION

Among malignancies, metastatic breast carcinoma is the second leading cause of death in woman [1]. Excessive and chronic activation of the immune system is involved in malignant transformation and metastasis. Cancer cells may also induce a chronic inflammatory response in the host, further enhancing the metastatic process by creating an inflammatory microenvironment [2]. CD200 is a widely expressed cell surface glycoprotein of an immunoglobulin family and inhibits inflammatory response [3] mainly through CD200 receptor (CD200R), which is expressed mostly by myeloid cells [4, 5], and some subsets of lymphoid-derived cells [4]. We previously documented that CD200 has a bidirectional effect on tumor growth and metastasis of breast carcinoma which depends on immunological response created by the tumor [6]. Specifically over expression of CD200 in the host reduced the progression and metastasis of highly aggressive and inflammatory 4THM murine breast carcinoma. These results suggest that CD200 mimetics may inhibit metastatic growth of tumor cells that induce systemic and local inflammatory response.

Soluble CD200 fusion protein, containing the ectodomain of CD200 bound to a murine IgG2a module (CD200fc) is used to mimic the effects of CD200 [7]. Specifically CD200fc markedly decreases the inflammatory response of various syndromes including infectious and autoimmune diseases and organ transplantation [4, 8–15]. To the best of our knowledge, the effects of CD200 mimetics on metastatic breast cancer are not known. Hence in the present study we aimed to evaluate the effects of CD200fc on visceral metastasis in an inflammatory, highly aggressive breast cancer model. Here we used 4THM cells which were obtained from the heart metastasis of 4T1 breast cancer cells. 4T1 cells were originally obtained from a spontaneously-formed breast cancer in a BALB/c mouse. We previously reported that 4THM cells comprise features of cancer stem cells and act more aggressively inducing systemic inflammatory response [16–18]. Here, we examined the effects of CD200 on tumor growth, metastasis, and anti-tumour immune responses to orthotopically-injected 4THM breast carcinoma cells using BALB/c female mice.

## RESULTS

### Growth of primary tumors and lung metastasis of 4THM breast carcinoma cells is attenuated by CD200fc

**Figure 1:**
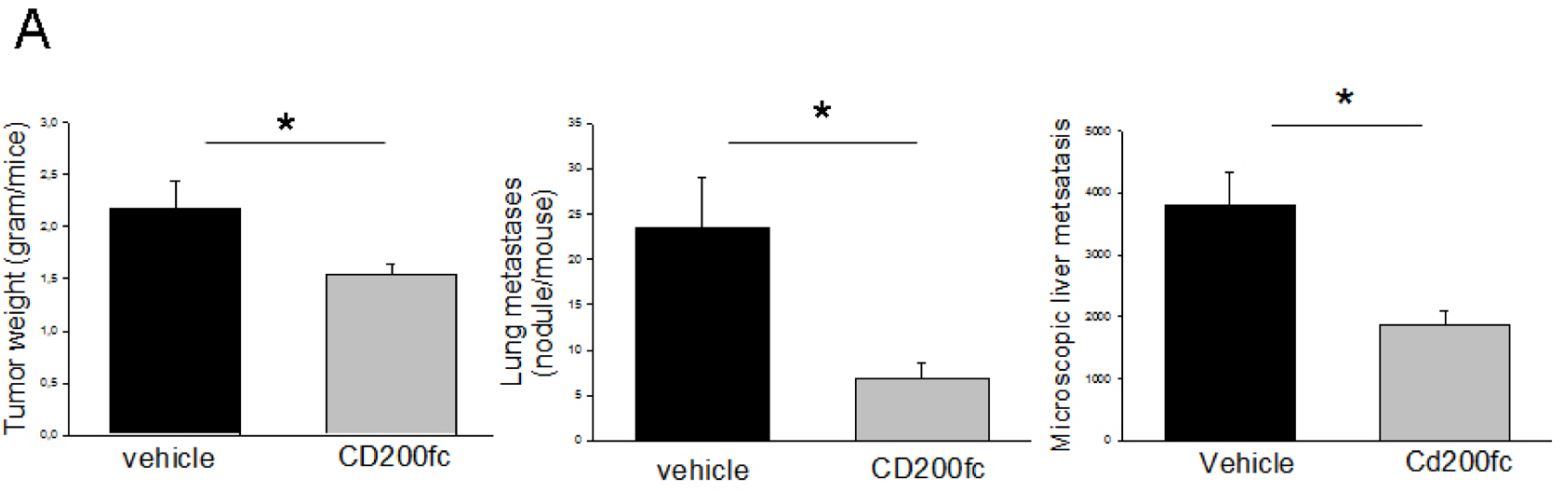

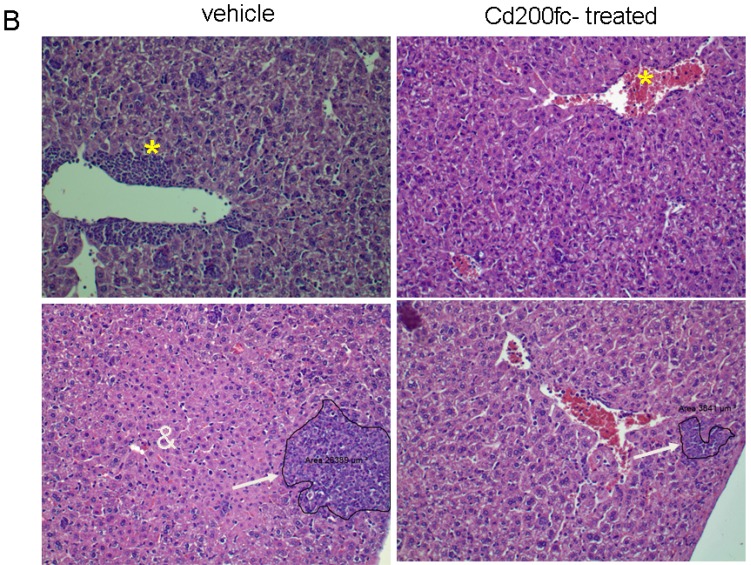
(**A**) Changes in tumor growth and metastasis of 4THM cells following CD200fc treatment. ^*^p < 0,05 (**B**) Microscopic appearance of liver metastasis and neutrophil infiltration (^*^).

Balb-c mice were injected with 4THM cells (10^5^ cells/mouse) orthotopically, and primary tumor growth and visceral metastases were evaluated 25 days after injection. As shown in Figure 1, growth of 4THM primary tumors were attenuated following treatment with CD200fc (100 μg/mouse) which were statistically significant. Lung metastases were also reduced significantly consistent with the altered pattern of primary tumor growth. Macroscopic nodules in the lungs were counted since they were clearly observed following fixation with Bouins solution and correlated with the microscopic results [18].

Liver metastases were evaluated microscopically and analyzed to determine the extent of metastases. As shown in Figure 1A and 1B, the degree of metastases was significantly decreased in CD200fc treated mice. We also observed that hepatocytes near metastatic lesions were degenerated whereas normal appearing hepatocytes were observed near metastatic lesions of CD200fc-treated mice. In accordance leukocyte extravasation was marked in untreated group but not in treated group (Panel B).

### CD200fc treatment inhibited 4THM-induced increases of Gr1+ cells and partly preserved CD200 expression in liver tissue

**Figure 2:**
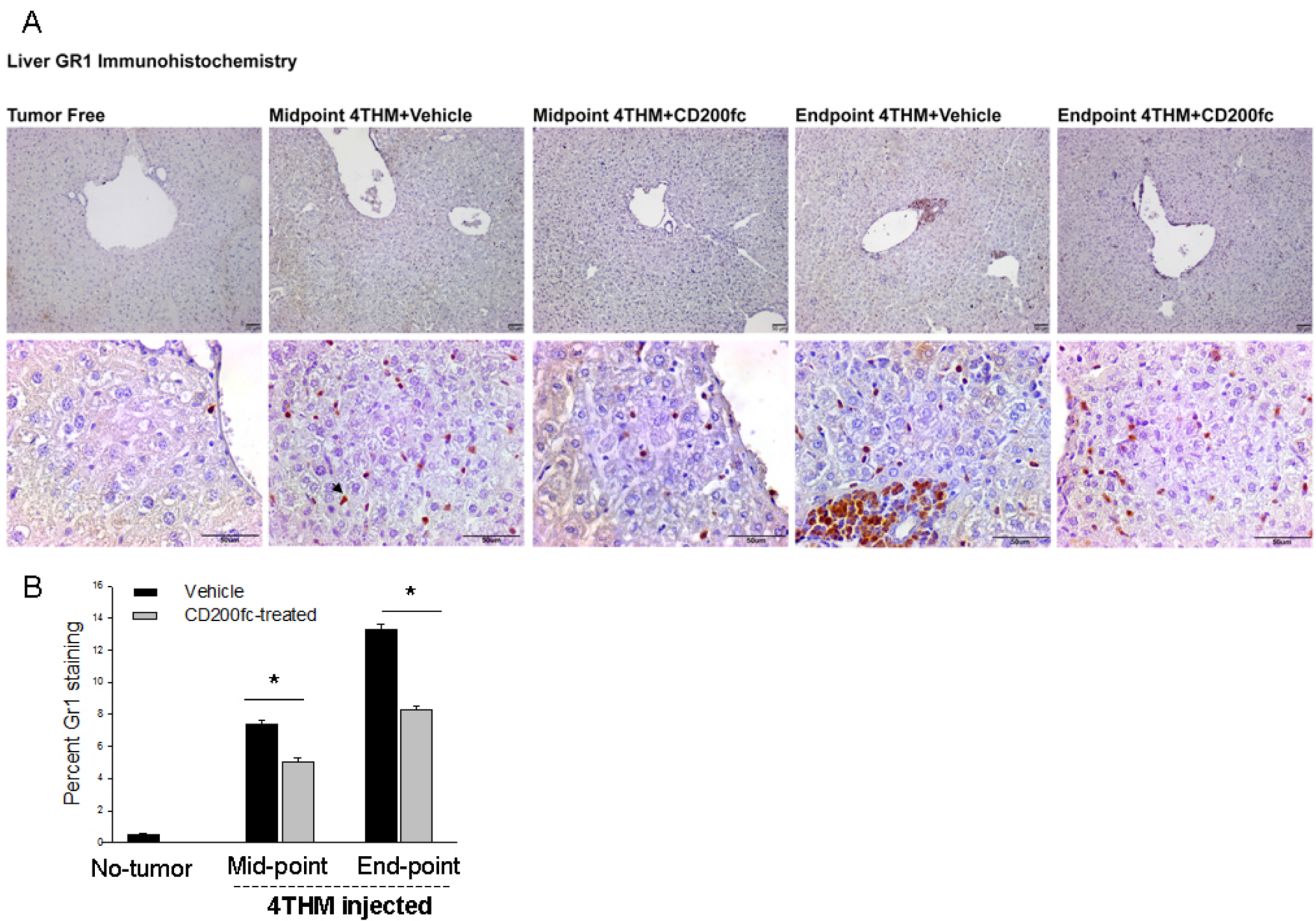

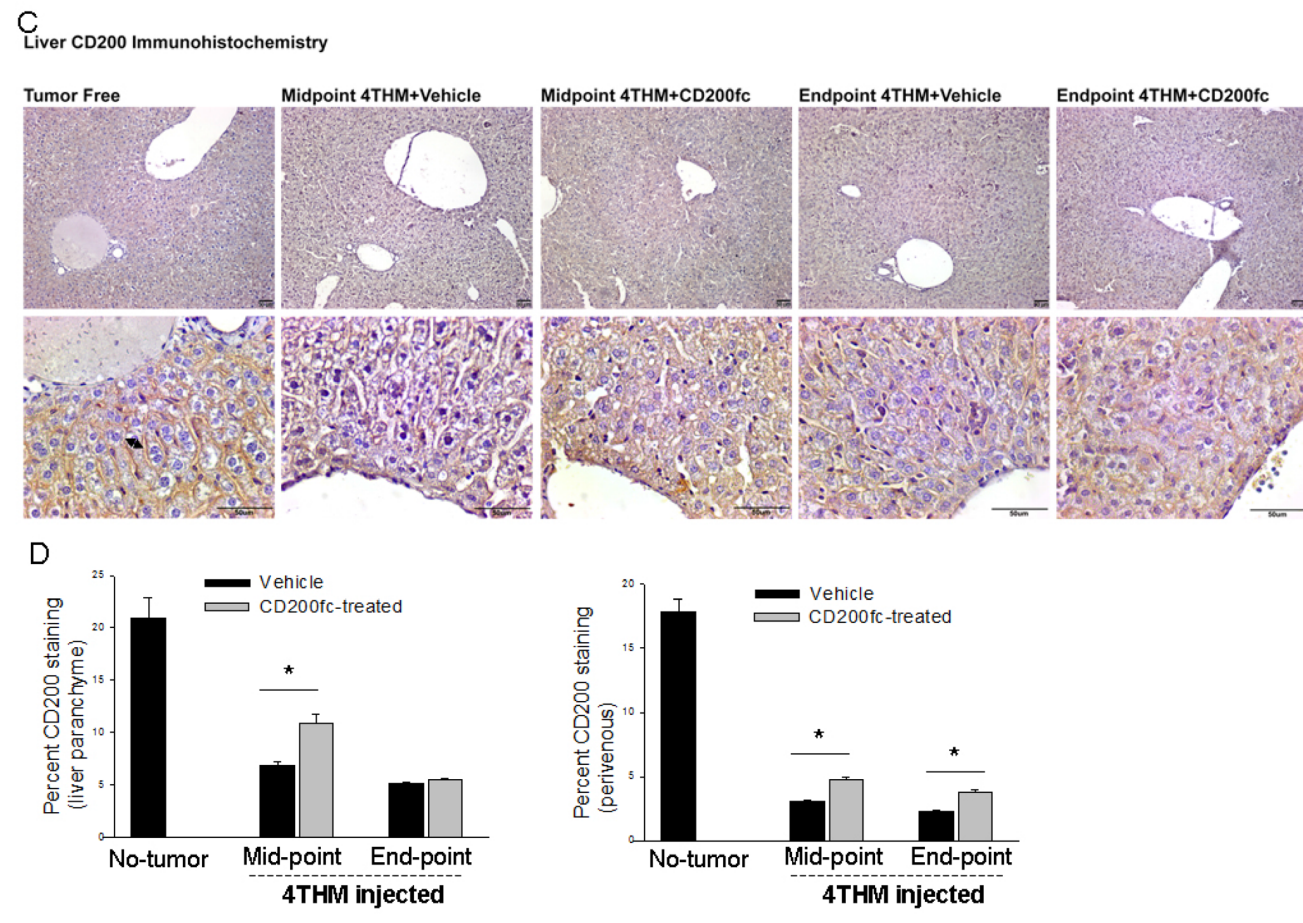
Changes in Gr1+ and CD200+ cells within liver tissue following CD200fc Treatment (**A**) Immunohistochemical staining of liver tissues with Gr1, arrow shows postive cells. (**B** and **D**) Image J analysis of the stainings, ^*^p < 0,05. (**C**) Immunohistochemical staining of liver tissues with Gr1, arrow heads show membrenous staining of hepatocytes.

To further verify type of immune cells extravasated, Gr1 staining in liver tissue was performed which indicates granulocytes (neutrophils) and may also demonstrate the homing of myeloid derived suppressor cells. There were remarkable differences in the extent of perivascular Gr1+ cells (extravasated) as well as Gr1+ cells invading the paranchyme in CD200fc treated group at both mid-point and the end-point (Figure 2A, 2B). Specifically CD200fc treatment significantly decreased Gr1+ cells in liver tissue of mice injected with 4THM cells.

Decreased neutrophil infiltration in CD200tg mice was reported before [6, 19]. We here also tested the possibility of alteration in CD200 expression of liver tissue. As seen in Figure 2C and 2D, 4THM tumors significantly decreased both perivenous and paranchymal expression of CD200 in liver tissue demonstrating that increased infiltration of granulocytes might be due to loss of CD200 expression. CD200fc treatment on the other hand partly preserved perivascular CD200 expression throughout the tumor growth. Hence CD200fc-induced decreases in liver metastasis might be partly due to preservation of endogenous CD200 activity.

### CD200 expression was increased in tumor and spleen tissue after treatment with CD200fc

**Figure 3:**
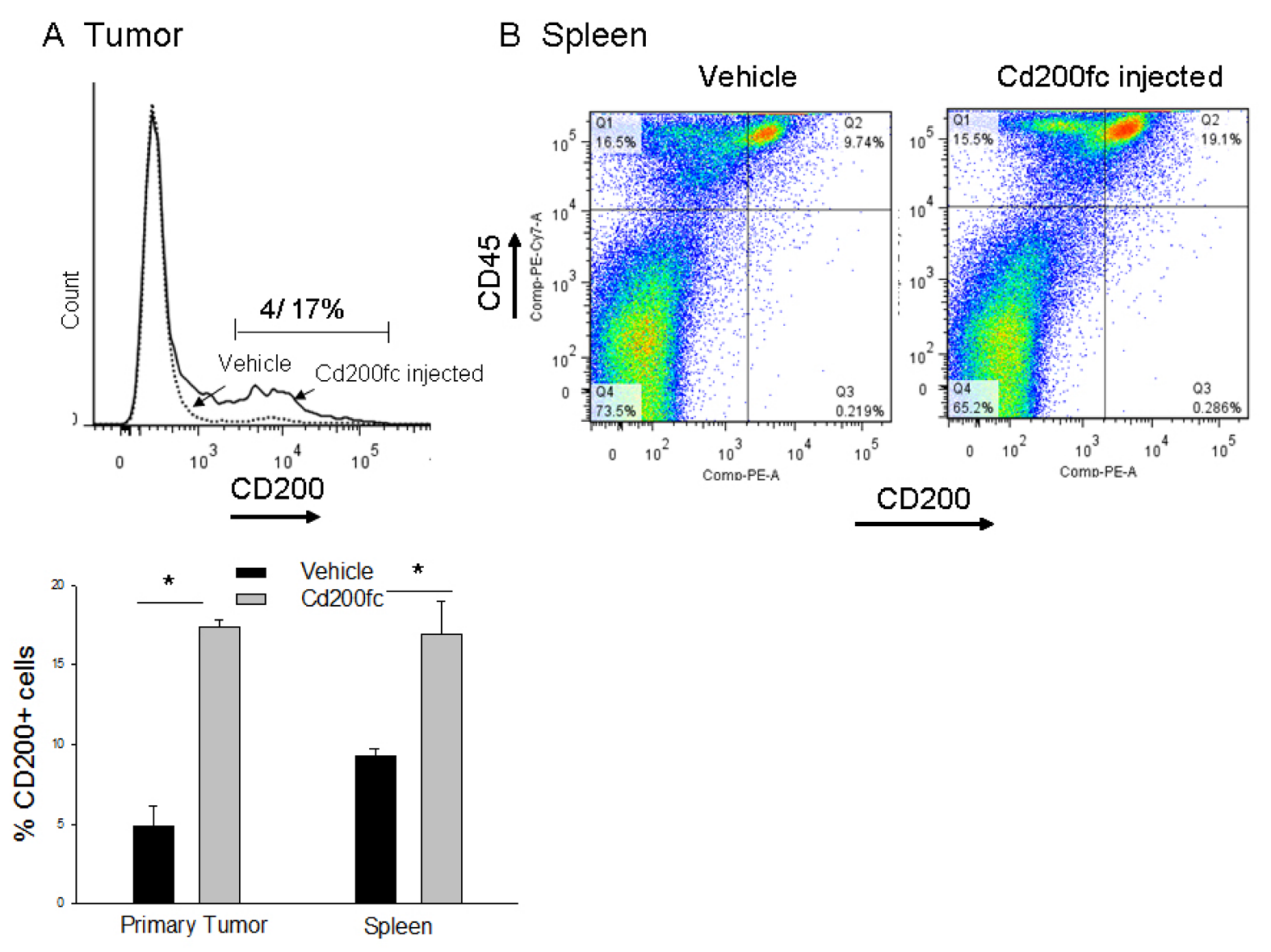
Flow cytometric analysis of CD200 positive cells within the primary tumor tissue and spleen (**A**) Changes in expression of CD200 on CD45^-^ cells within tumor tissue following CD200fc treatment, ^*^p < 0,05. (**B**) Changes in expression of CD45 and CD200+ cells of spleen following CD200fc treatment.

Previously using flow cytometry and IHC, we showed that 4THM cells as well as metastatic tumors originated from 4THM primary tumors do not express CD200 [6]. In accordance we observed that in the untreated group only around 4% of CD45- cells were expressing CD200 indicating presence of CD200 on non-tumoral cells e.g. stromal cells. Membrane expression of CD200 was significantly increased in CD45- cells in CD200fc-treated mice demonstrated by flow cytometry. Similarly CD200 levels on splenocytes were significantly higher in CD200fc-treated group (Figure 3). These results were in accordance with previous ones demonstrated in the liver tissue. Increased CD200 levels in spleen seemed to alter splenic CD200R1 expression which was significantly decreased following CD200fc treatment (Supplementary Figure 1).

### Flow cytometric analysis of CD3, CD8, CD4, Gr-1, CD11b expression by tumor infiltrating immune cells and spleen

**Figure 4:**
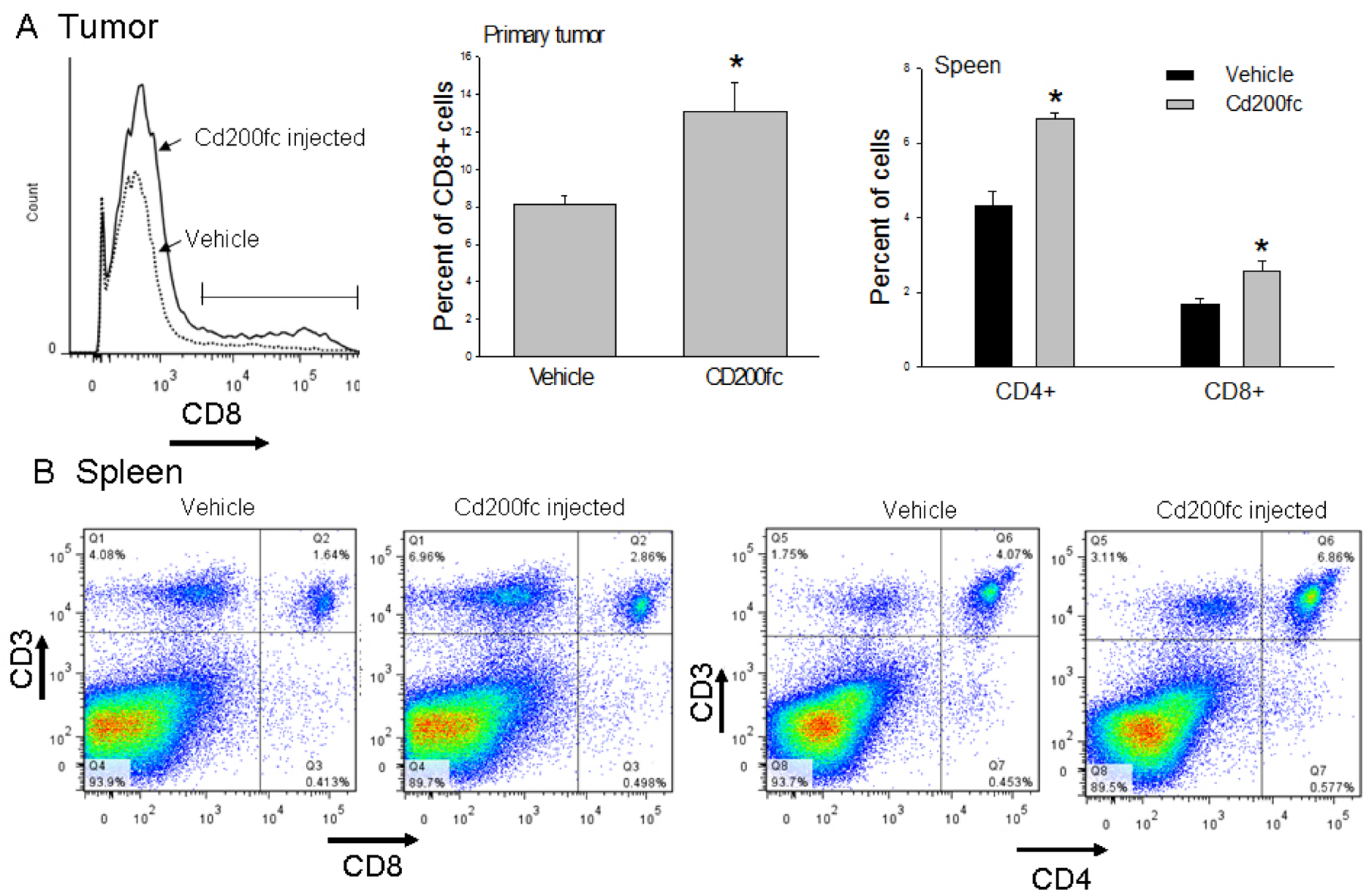
Flow cytometric analysis of CD8+ and CD4+ cells (**A**) Effects of CD200fc treatment on tumor infiltrating CD8+ cells. (**B**) Effects of CD200fc treatment on the levels of Cd3+Cd4+ and CD3+CD8+ cells of the spleen tissue. ^*^p < 0,05 compared to vehicle.

Tumor infiltrating immune cells were evaluated both at the mid-point (12 days after 4THM injection; animals received 3 injections of CD200fc) and at the end point (25 day after 4THM injection). Differential changes were observed in CD8+ cells which were significantly increased at the end point following CD200fc treatment. Similar changes were also observed earlier in spleen (mid-point) such that CD200fc treatment significantly increased the number of CD8+ cells. In addition CD200fc-treatment also increased splenic CD4+ cells 12 days after injection of tumor cells. This time-dependent changes indicate that CD200fc first increases T cell number systemically and the cytotoxic T-cells eventually infiltrate tumor tissue (Figure 4).

CD11b+Gr1+ cells in draining lymph nodes and spleen were significantly decreased following CD200fc treatment (Supplementary Figure 2).

### CD200fc increased IFN-γ responses and time-dependently altered IL-10 secretion

MLCs were prepared 12 and 25 days after 4THM injection, as described in *Methods*. Day 12 was chosen, because 4THM cells grow sufficiently to start metastasizing by this time and; day 25 MLCs were used to assess changes in the immune response in the presence of extensive disease.

**Figure 5:**
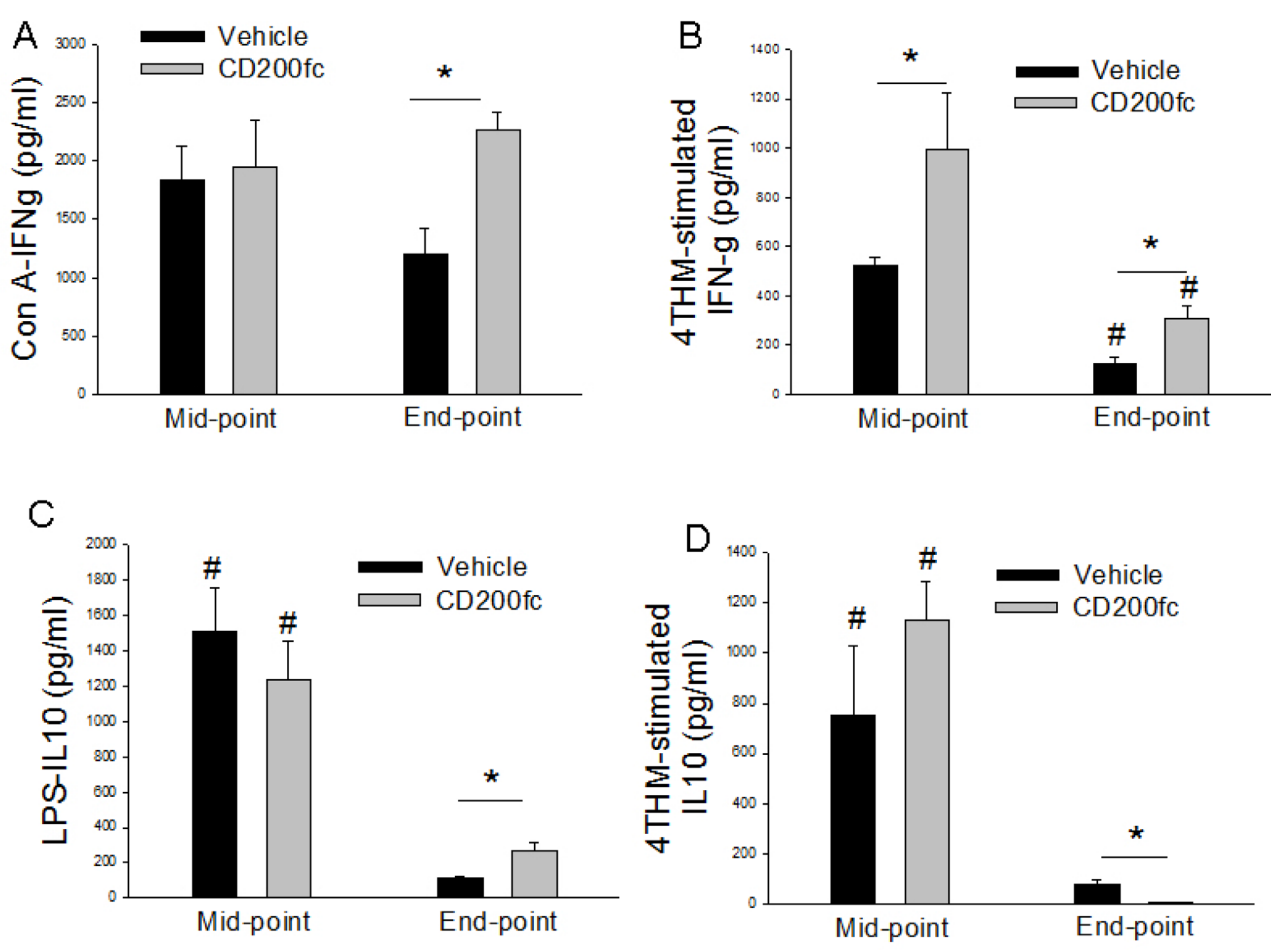
IFN-g (**A, B**) and IL-10 (**C, D**) secretion from MLCs prepared from vehicle of CD200fc-treated mice bearing 4THM tumors for 12 (midpoint) and 25 (endpoint) days. Con-A- MLCs stimulated with concavalin A for 40 h; 4THM- MLCs stimulated with irradiated 4THM cells for 40 h. LPS- MLCs stimulated with lipopolysaccaride for 40 h; 4THM. ^*^p < 0,05 significantly different from corresponding WT MLCs. ^*^p < 0,05; ^#^*p* < 0,05 compared to end-point or mid-point.

The irradiated 4THM-induced IFN-γ response was significantly higher in MLCs of CD200fc-treated mice compared with untreated group at both end-point and the mid-point (Figure 5). This was similar to the effects observed in CD200tg animals. CD200fc increased IFN-γ response to Con-A 25 days after 4THM injection which was markedly lower compared to mid-point.

In terms of IL-10 secretion, there was a clear difference between end-point and mid-point. Specifically both LPS and irradiated 4THM-induced IL-10 response was significantly attenuated at the end-point. Interestingly, CD200fc treatment enhanced IL-10 secretion after LPS challenge and suppressed after 4THM challenge at the end-point (Figure 5).

### CD200fc decreased IL-6 secretion and time-dependently altered IL-17 response

**Figure 6:**
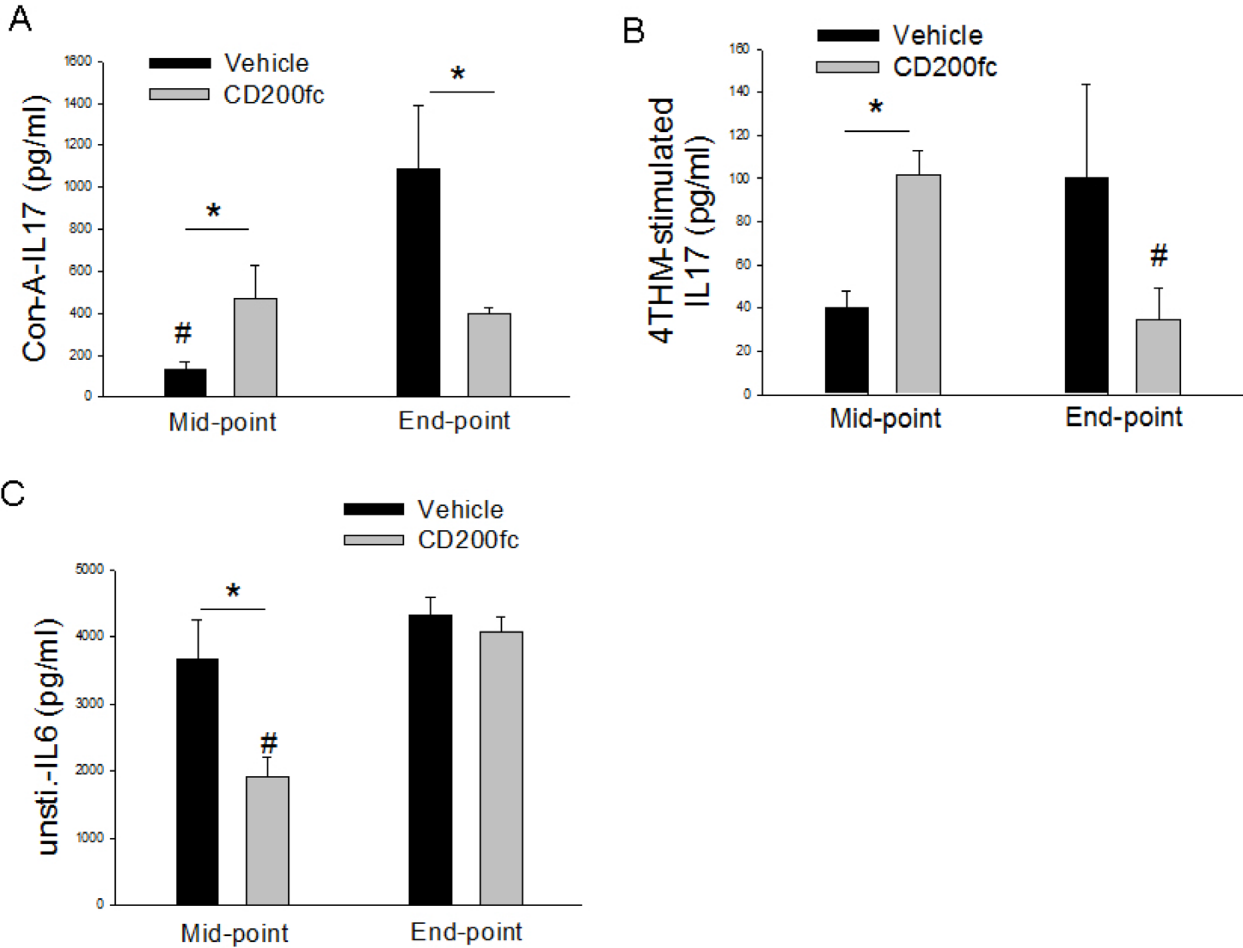
IL-17 (**A, B**) and IL-6 (**C**) secretion from MLCs prepared from vehicle of CD200fc-treated mice bearing 4THM tumors for 12 (midpoint) and 25 (endpoint) days. Con-A- MLCs stimulated with concavalin A for 40 h; 4THM- MLCs stimulated with irradiated 4THM cells for 40 h. Unsti-unstimulated; TNF-a secretion were measured 20 h after incubation of MLC. ^*^p < 0,05 significantly different from corresponding WT MLCs. ^*^p < 0,05; ^#^*p* < 0,05 compared to end-point or mid-point.

MLC obtained from tumor-bearing animals secreted large amount of IL-6 even in the absence of a challenge. CD200fc treatment significantly suppressed basal IL-6 secretion at the mid-point correlating its known anti-inflammatory effects. This effect of CD200fc on the other hand was not present at the end-point (Figure 6).

Bidirectional effects of CD200fc on IL-17 response to Con-A and irradiated tumor cells were observed. Specifically CD200fc treatment increased IL-17 response to Con-A and irradiated tumor cells at the mid-point while the opposite was observed at the end-point (Figure 6).

## DISCUSSION

Possible therapeutic value of CD200 fusion protein (CD200fc) was not previously studied in an aggressive breast carcinoma model under *in-vivo* condition. 4THM breast carcinoma cells create a cancer model in an immunocompetent syngeneic animal which mimics treatment resistant grade 4 breast carcinoma. Specifically these cells demonstrate cancer stem cell phenotype [18], and induce extensive visceral metastasis. We here reported that CD200fc markedly inhibited lung and liver metastasis of 4THM cells inoculated orthotopically demonstrating therapeutic value of CD200fc in aggressive breast carcinoma. The effect of CD200fc on primary tumor growth, although significant, was limited which is not surprising given the fact that these cells do not express CD200R [6]. This also supports our findings suggesting that increased anti-tumoral immune response mediate suppressive effects of CD200fc treatment on visceral metastasis.

CD200fc, a CD200R1 agonist, has been found to have anti-inflammatory effects in neurodegenerative and autoimmune diseases [10, 12, 20–23]. Up to our knowledge possible therapeutic effects of CD200fc was not previously evaluated in a cancer model under *in-vivo* conditions. Recently He *et al.* demonstrated that CD200fc inhibits LPS-induced IL-1b secretion from cervical cancer cells which contributes to the pathogenesis of HPV-infected cervical carcinoma [24, 25]. Hence authors concluded that CD200fc may inhibit cervical cancer formation. Direct evidence, however was lacking and we here showed that systemic treatment with CD200fc markedly suppresses systemic metastasis of breast carcinoma for the first time. These findings are in accordance with our previous findings documenting marked suppression of 4THM-induced tumor growth and metastasis in mice over expressing CD200 (CD200tg) [6].

Cancer-related inflammation is a hallmark of cancer [26] and in many cases is increased in parallel with tumor growth [27]. Although CD200 is known to be immunosuppressive, its potent anti-inflammatory effects dominate in inflammation driven carcinogenesis for which 4THM is a model. CD200fc mimicked the effects of endogenously overexpressed CD200 and inhibited tissue infiltration of neutrophils. Furthermore mix leukocyte culture obtained from CD200fc-treated tumor-bearing mice secreted markedly lower levels of IL-6. CD200fc also inhibited LPS-induced IL-6 secretion from microglia [28] correlating with our findings. Hence our results further emphasize that anti-inflammatory effects of CD200fc play an important role in its anti-tumoral effects. Given that the degree of inflammation correlates with the degree of aggressiveness and treatment resistance in most carcinomas, CD200fc may thus exert potent anti-tumoral and anti metastatic effects.

We previously observed that lack of CD200R1 expression resulted in significantly increased lung and liver metastasis of 4THM tumors [6] hence anti-tumoral effects of CD200fc may largely be due to the activation of CD200R1.

Intra-tumoral CD8^+^ T-cell infiltration is associated with delayed recurrence and extended survival in patients [29], and infiltration of breast cancer with CD8^+^ cytotoxic T lymphocytes is associated with a good response to chemotherapy [30]. IFN-γ is produced mainly by natural killer (NK) cells and specific T-cell subsets that play a critical role in an antitumoral immune response [31]. IFN-γ induces activation of effector macrophages, which can lyse the tumor cells, increase antigen presentation, and inhibit angiogenesis [32]. CD200fc increased tumor infiltrating CD8+ T cell number and increased tumor-induced IFN-γ secretion at both mid and end-point demonstrating enhancement of anti-tumoral immunity. These effects likely to be mediated by CD200R1 receptors since lack of CD200R1 expression by the host was associated with decreased numbers of tumor infiltrating CD8^+^ T cells in the same model [6]. We previously observed that CD200 overexpression by CD200tg hosts resulted in increased tumor-induced IFN-γ and decreased IL-6 responses further documenting efficiency of CD200fc as CD200 mimetic.

4THM cells are not only very aggressive, but they also include many properties of cancer stem cells (CSCs) [18]. Recent studies demonstrated that CSCs are involved in induction of local and systemic inflammation [33]. Breast CSCs have been reported to express inflammatory chemokines, as well as inflammatory cytokines, including TNF-α and IL-6. 4THM cells secrete high levels of MIP-2, the chemokine involved in neutrophil chemotaxis and angiogenesis [18]. IL-6 is one of the main mediators of inflammation-induced stemness in breast cancer [34, 35]. Furthermore clinical studies demonstrated increased serum concentrations of IL-6 in breast cancer patients are strongly associated with tumor stage and poor prognosis [36]. Hence decreases in IL-6 secretion in CD200fc-treated mice likely to contribute observed anti-metastatic effects of CD200fc treatment.

IL-10 can be secreted by both Th1 and Th2 cells and has a dual role on tumor development and carcinogenesis [37]. IL-10 acts as an anti-inflammatory molecule preventing tumor development in animal models of chronic inflammation-induced carcinogenesis [38], and inducing regression of established breast cancer metastases [39]. Similarly, in our inflammatory model, IL-10 secretion markedly decreased at the advanced disease compared to earlier time points which was also reported before [6]. On the other hand, IL-10 has immunosuppresive effects and also is associated with tumor progression such that tumor IL-10 levels correlate with disease severity [37]. Interestingly, CD200fc differentially altered IL-10 response to LPS and irradiated tumor challenge. Specifically CD200fc was able to increase IL-10 response to LPS challenge but markedly inhibited IL-10 response to 4THM cells at the end point. These findings correlates with the observed anti-tumoral effects, such that immunosuppressive effects of IL-10 within tumor microenvironment was abolished while systemic anti-inflammatory effects of IL-10 was enhanced by CD200fc treatment. These effects of CD200fc were different from the ones observed in the presence of endogenously over expressed CD200 demonstrating that CD200fc may also have distinct consequences [6].

Different studies have shown that IL-17 can favor or counteract tumor growth, depending on the tumor type, type of tumor infiltrating immune cells and the balance of other factors in the microenvironment [40–42]. IL-17 may increase aggressiveness of tumor cells by directly acting on cancer cell as well as by inducing angiogenesis and immune suppression [43]. On the other hand IL-17 stimulates dendritic cells (DCs) to produce IL-12, while inhibiting IL-10 to augment Th1 differentiation [44, 45]. IL-17 expressed by Th17 cells is important for initiating this immune reaction and eventually leads to activation of protective tumor-specific IFN-γ expressing CD8 T cells [43]. IL-17 is secreted from different cell types including neutrophils, macrophages, mast cells and Th17 cells [40]. It was recently shown that, increased levels of IL17 secreting neutrophils correlates with poor prognosis while increased Th17 cells is an independent prognostic factor for improved survival in cervical cancer [40].

The effects of CD200fc treatment varied time-dependently. Specifically IL-17 response to Con-A and irrediated tumor cells were higher in CD200fc treatment group at earlier stage of the disease (12 days after injection of tumor cells) but lower at the final stage of the carcinoma (25 days after injection of tumor cells). Time-dependent changes in immune cell type systemically and locally are likely to be responsible from bidirectional effect of CD200fc observed here. Neutrophil count increases systemically and within tumor tissue during final stage of the disease in 4THM breast cancer model [6]. Specifically we observed that primary tumors became necrotic, most of the cells obtained from tumor digestion were granulocytes (64%) in the terminal stages of tumor growth. Similarly peripheral blood smears documented approximately more than 20 times increases in neutrophils [6, 18, 46]. We also observed that tumor-infiltrating T cell count as well as number of T-cells in draining lymph nodes and spleen is much higher 12 days compared to 25 days after inoculation of tumor cells (unpublished observations). Hence our results with previously published data suggest that CD200fc decreases IL-17 secretion from neutrophils preventing tumor-promoting effects of neutrophils while enhances IL-17 secretion from T cells presumably enhancing tumorocidal immune response. In accordance inhibition of myeloid cells by CD200 was reported before [47, 48]. Whether these observed effects are direct or not require further studies. Previous studies however suggest the observed effects are indirect. Specifically CD200fc does not directly suppress T cell responses but acts primarily on the inflammatory response [49]. Similarly CD200-CD200R1 activation not only decreases the production of proinflammatory cytokines, but enhances the production of anti-inflammatory cytokines [28].

4THM tumor model mimics many aspects of treatment resistant breast carcinoma, including the presence of breast CSCs, epithelial mesenchymal transition, extensive visceral metastases, and an undifferentiated state [18]. Our results in combination with previous studies demonstrated that CD200fc is effective for the treatment of breast tumors, especially in those characterized by increased systemic and local inflammatory response. Decreased inflammation and enhanced anti-tumoral immune responses are likely to underlie the anti-tumoral effects of CD200fc.

## MATERIALS AND METHODS

### Mice

Wild-type (WT) female BALB/c mice were purchased from the Jackson Laboratories, Bar Harbor, ME. Mice were housed 5 per cage under specific pathogen-free conditions and allowed food and water *ad libitum*. All mice were used at 8–12 wk of age. Study was approved by University Health Network, Animal Care Committee, Toronto General Hospital and all animal experimentation was performed following the guidelines of an accredited animal care committee.

### CD200fc treatment

CD200 was cloned from mouse and raised in CHO cells. Animals received CD200fc dissolved in PBS (20 μg/mouse, iv, 0.2 ml) two times a week for four times. Following forth injection we observed an anaphylactoid-like reaction in four of the tumor-bearing animals which died within 30 min, hence fifth injection was given i.p. in 0.2 ml (total dose was 100 μg/mouse). We believed that this anaphylactoid-like reaction precipitated because of systemic inflammation occurred around the time of 4. injection (15 days after injection of tumor cells). Necropsies were performed 12 and 25 days after injection of 4THM cells.

### Cell line

4T1 breast cancer cells were previously derived from a spontaneously appearing breast tumor in a BALB/c female mouse. The 4THM cell line was derived from cardiac metastases of 4T1 cells by Erin *et al.* [50, 51]. 4THM cells were grown in α-MEM (Thermofisher, cat no: 12561056) supplemented with Penicillin and streptomycin and 5% FBS.

### Metastasis assay

4THM cells (1 × 10^5^ cells per mouse) were injected orthotopically into the right upper mammary gland of 8–10 wk female recipients. Necropsies were performed either 12 or 25 days after injection, with lung tissues stored in Bouin’s fixative to visualize macroscopic nodules, as described before [17, 50]. Liver tissue was originally fixed in 10% buffered formalin and sectioned after embedding in paraffin wax. 5 sections from each tissue were stained with hematoxylin and eosin (H&E) to determine the extent of metastases microscopically. For each animal, 20 photographs from 5 different sections were randomly taken at 20× magnification; areas of microscopic metastatic lesions were then selected and measured as mm^2^ with Spot A dvanced 4.6 (Diagnostic Instruments, Inc, Michigan, USA) software.

### Mixed leukocyte cultures

Spleen and draining lymph nodes of animals injected with 4THM cells were removed aseptically and single cell suspensions were prepared for mixed leukocyte cultures (MLCs) in RPMI medium 1640 (Gibco). Cells (4 × 10^6^/well in 48-well tissue culture plate) were cultured alone (control for basal cytokine release) or stimulated with LPS 3 μg/ml or irradiated (20 Gray) 5 × 10^4^ 4THM cells. MLCs from control animals (not injected with 4THM cell) were also prepared. TNF-α levels were measured 20 hrs after challenge, while measurements of other cytokines were performed in supernatants obtained 40 hrs after challenge. Standard ELISA kits were used for cytokine measurements (all obtained from Biolegend).

### Antibodies and cell staining

All anti-mouse monoclonal antibodies used for cell-surface phenotype characterization were purchased from BioLegend. Multicolour flow cytometric analyses were conducted to characterize draining lymph node cells, splenocytes, and tumor infiltrating cells. Tumors, draining lymph nodes, and spleens were digested as described before [52]. The optimal concentration of antibody for staining was titrated individually for each antibody. Single color controls were included in each experiment for compensation purposes, as well as fluorescence-minus-one (FMO) controls; all samples were analyzed in a CANTO-II flow cytometer, using FloJo software. Samples from 2–3 mice were pooled per staining group and staining was performed in triplicate.

### Immunohistochemistry

Liver and spleen tissues were fixed in 10% formalin and embedded in paraffin. 5 μm thick serial sections were collected. Staining with heterologous rabbit antibodies for mouse CD200 (prepared after immunization in Freund’s adjuvant with protein purified from supernatants of CHO cells transduced to express the respective cloned genes: Gorczynski *et al.* unpublished), GR-1 antibody (1/400 dilution) was from Abcam (ab25377) and stainings were performed essentially as described elsewhere [18].

Immunohistochemical images of all tissue samples were captured using Spot Imaging software version 4.6 (Diagnostic Instruments, Inc, Michigan, USA) at 200 magnification. Ten photomicrographs were randomly selected for each group and analyzed by Image-J Version 1.46 (National Institutes of Health, Bethesda, Maryland, USA). All tissue components except background were measured by moving brightness slider until all stained areas were selected and recorded to the excel sheet. To measure the stained areas only, the hue slider was decreased without changing the brightness slider, until only the immunohistochemistry (IHC) stained areas were selected and recorded, as well. Finally, integrated density values of IHC stained areas were normalized to the integrated density values of the total area. The calculated ratio of the immunostained area to total area was graphed.

### Statistics

Student’s *t* test was used to compare metastatic indexes. When the variance expressed as standard deviation (SD) between the groups differed, the Welch *t* test or non-parametric (Mann–Whitney test) was used. ANOVA with Dunnett’s post test was used for the analysis of cytokine results as well results of Image-J analysis. *P* values < 0.05 were considered biologically significant. Statistical analyses were performed using GraphPad InStat 3 software.

## SUPPLEMENTARY MATERIALS FIGURES



## References

[1] DeSantis CE, Fedewa SA, Goding SA, Kramer JL, Smith RA, Jemal A (2016). Breast cancer statistics, 2015: convergence of incidence rates between black and white women. CA Cancer J Clin.

[2] Hanahan D, Weinberg RA (2011). Hallmarks of cancer: the next generation. Cell.

[3] Wright GJ, Puklavec MJ, Willis AC, Hoek RM, Sedgwick JD, Brown MH, Barclay AN (2000). Lymphoid/neuronal cell surface OX2 glycoprotein recognizes a novel receptor on macrophages implicated in the control of their function. Immunity.

[4] Gorczynski R, Chen Z, Kai Y, Lee L, Wong S, Marsden PA (2004). CD200 is a ligand for all members of the CD200R family of immunoregulatory molecules. J Immunol.

[5] Wright GJ, Cherwinski H, Foster-Cuevas M, Brooke G, Puklavec MJ, Bigler M, Song Y, Jenmalm M, Gorman D, McClanahan T, Liu MR, Brown MH, Sedgwick JD (2003). Characterization of the CD200 receptor family in mice and humans and their interactions with CD200. J Immunol.

[6] Erin N, Podnos A, Tanriover G, Duymus O, Cote E, Khatri I, Gorczynski RM (2015). Bidirectional effect of CD200 on breast cancer development and metastasis, with ultimate outcome determined by tumor aggressiveness and a cancer-induced inflammatory response. Oncogene.

[7] Gorczynski RM, Cattral MS, Chen Z, Hu J, Lei J, Min WP, Yu G, Ni J (1999). An immunoadhesin incorporating the molecule OX-2 is a potent immunosuppressant that prolongs allo- and xenograft survival. J Immunol.

[8] Gorczynski RM, Chen Z, He W, Khatri I, Sun Y, Yu K, Boudakov I (2009). Expression of a CD200 transgene is necessary for induction but not maintenance of tolerance to cardiac and skin allografts. J Immunol.

[9] Boudakov I, Liu J, Fan N, Gulay P, Wong K, Gorczynski RM (2007). Mice lacking CD200R1 show absence of suppression of lipopolysaccharide-induced tumor necrosis factor-alpha and mixed leukocyte culture responses by CD200. Transplantation.

[10] Hernangomez M, Carrillo-Salinas FJ, Mecha M, Correa F, Mestre L, Loria F, Feliu A, Docagne F, Guaza C (2014). Brain innate immunity in the regulation of neuroinflammation: therapeutic strategies by modulating CD200-CD200R interaction involve the cannabinoid system. Curr Pharm Des.

[11] Lynch MA (2014). The impact of neuroimmune changes on development of amyloid pathology; relevance to Alzheimer's disease. Immunology.

[12] Hernangomez M, Mestre L, Correa FG, Loria F, Mecha M, Inigo PM, Docagne F, Williams RO, Borrell J, Guaza C (2012). CD200-CD200R1 interaction contributes to neuroprotective effects of anandamide on experimentally induced inflammation. Glia.

[13] Jenmalm MC, Cherwinski H, Bowman EP, Phillips JH, Sedgwick JD (2006). Regulation of myeloid cell function through the CD200 receptor. J Immunol.

[14] Zhang S, Cherwinski H, Sedgwick JD, Phillips JH (2004). Molecular mechanisms of CD200 inhibition of mast cell activation. J Immunol.

[15] Snelgrove RJ, Goulding J, Didierlaurent AM, Lyonga D, Vekaria S, Edwards L, Gwyer E, Sedgwick JD, Barclay AN, Hussell T (2008). A critical function for CD200 in lung immune homeostasis and the severity of influenza infection. Nat Immunol.

[16] Erin N, Akdas BG, Harms JF, Clawson GA (2008). Vagotomy enhances experimental metastases of 4THMpc breast cancer cells and alters substance P level. Regul Pept.

[17] Erin N, Wang N, Xin P, Bui V, Weisz J, Barkan GA, Zhao W, Shearer D, Clawson GA (2009). Altered gene expression in breast cancer liver metastases. Int J Cancer.

[18] Erin N, Kale S, Tanriover G, Koksoy S, Duymus O, Korcum AF (2013). Differential characteristics of heart, liver, and brain metastatic subsets of murine breast carcinoma. Breast Cancer Res Treat.

[19] Erin N, Boyer PJ, Bonneau RH, Clawson GA, Welch DR (2004). Capsaicin-mediated denervation of sensory neurons promotes mammary tumor metastasis to lung and heart. Anticancer Res.

[20] Erin N, Zhao W, Bylander J, Chase G, Clawson G (2006). Capsaicin-induced inactivation of sensory neurons promotes a more aggressive gene expression phenotype in breast cancer cells. Breast Cancer Res Treat.

[21] Gorczynski RM, Chen Z, Diao J, Khatri I, Wong K, Yu K, Behnke J (2010). Breast cancer cell CD200 expression regulates immune response to EMT6 tumor cells in mice. Breast Cancer Res Treat.

[22] Chen Z, Yu K, Zhu F, Gorczynski R (2016). Over-Expression of CD200 Protects Mice from Dextran Sodium Sulfate Induced Colitis. PLoS One.

[23] Hernangomez M, Klusakova I, Joukal M, Hradilova-Svizenska I, Guaza C, Dubovy P (2016). CD200R1 agonist attenuates glial activation, inflammatory reactions, and hypersensitivity immediately after its intrathecal application in a rat neuropathic pain model. J Neuroinflammation.

[24] Lyons A, Downer EJ, Costello DA, Murphy N, Lynch MA (2012). Dok2 mediates the CD200Fc attenuation of Abeta-induced changes in glia. J Neuroinflammation.

[25] Jiang L, Xu F, He W, Chen L, Zhong H, Wu Y, Zeng S, Li L, Li M (2016). CD200Fc reduces TLR4-mediated inflammatory responses in LPS-induced rat primary microglial cells via inhibition of the NF-kappaB pathway. Inflamm Res.

[26] Ding Y, Yang H, Xiang W, He X, Liao W, Yi Z (2015). CD200R1 agonist attenuates LPS-induced inflammatory response in human renal proximal tubular epithelial cells by regulating TLR4-MyD88-TAK1-mediated NF-kappaB and MAPK pathway. Biochem Biophys Res Commun.

[27] He A, Shao J, Zhang Y, Lu H, Wu Z, Xu Y (2017). CD200Fc reduces LPS-induced IL-1beta activation in human cervical cancer cells by modulating TLR4-NF-kappaB and NLRP3 inflammasome pathway. Oncotarget.

[28] Dutta S, Chakraborty C, Mandal RK, Basu P, Biswas J, Roychoudhury S, Panda CK (2015). Persistent HPV16/18 infection in Indian women with the A-allele (rs6457617) of HLA-DQB1 and T-allele (rs16944) of IL-1beta -511 is associated with development of cervical carcinoma. Cancer Immunol Immunother.

[29] Colotta F, Allavena P, Sica A, Garlanda C, Mantovani A (2009). Cancer-related inflammation, the seventh hallmark of cancer: links to genetic instability. Carcinogenesis.

[30] Dvorak HF (1986). Tumors: wounds that do not heal. Similarities between tumor stroma generation and wound healing. N Engl J Med.

[31] Cox FF, Carney D, Miller AM, Lynch MA (2012). CD200 fusion protein decreases microglial activation in the hippocampus of aged rats. Brain Behav Immun.

[32] Zhang L, Conejo-Garcia JR, Katsaros D, Gimotty PA, Massobrio M, Regnani G, Makrigiannakis A, Gray H, Schlienger K, Liebman MN, Rubin SC, Coukos G (2003). Intratumoral T cells, recurrence, and survival in epithelial ovarian cancer. N Engl J Med.

[33] Seo AN, Lee HJ, Kim EJ, Kim HJ, Jang MH, Lee HE, Kim YJ, Kim JH, Park SY (2013). Tumour-infiltrating CD8+ lymphocytes as an independent predictive factor for pathological complete response to primary systemic therapy in breast cancer. Br J Cancer.

[34] Street SE, Trapani JA, MacGregor D, Smyth MJ (2002). Suppression of lymphoma and epithelial malignancies effected by interferon gamma. J Exp Med.

[35] Corthay A, Skovseth DK, Lundin KU, Rosjo E, Omholt H, Hofgaard PO, Haraldsen G, Bogen B (2005). Primary antitumor immune response mediated by CD4+ T cells. Immunity.

[36] Charafe-Jauffret E, Ginestier C, Iovino F, Wicinski J, Cervera N, Finetti P, Hur MH, Diebel ME, Monville F, Dutcher J, Brown M, Viens P, Xerri L (2009). Breast cancer cell lines contain functional cancer stem cells with metastatic capacity and a distinct molecular signature. Cancer Res.

[37] Sansone P, Storci G, Tavolari S, Guarnieri T, Giovannini C, Taffurelli M, Ceccarelli C, Santini D, Paterini P, Marcu KB, Chieco P, Bonafe M (2007). IL-6 triggers malignant features in mammospheres from human ductal breast carcinoma and normal mammary gland. J Clin Invest.

[38] Iliopoulos D, Hirsch HA, Struhl K (2009). An epigenetic switch involving NF-kappaB, Lin28, Let-7 MicroRNA, and IL6 links inflammation to cell transformation. Cell.

[39] Lippitz BE (2013). Cytokine patterns in patients with cancer: a systematic review. Lancet Oncol.

[40] Mosser DM, Zhang X (2008). Interleukin-10: new perspectives on an old cytokine. Immunol Rev.

[41] Erdman SE, Rao VP, Poutahidis T, Ihrig MM, Ge Z, Feng Y, Tomczak M, Rogers AB, Horwitz BH, Fox JG (2003). CD4(+)CD25(+) regulatory lymphocytes require interleukin 10 to interrupt colon carcinogenesis in mice. Cancer Res.

[42] Kundu N, Beaty TL, Jackson MJ, Fulton AM (1996). Antimetastatic and antitumor activities of interleukin 10 in a murine model of breast cancer. J Natl Cancer Inst.

[43] Punt S, Fleuren GJ, Kritikou E, Lubberts E, Trimbos JB, Jordanova ES, Gorter A (2015). Angels and demons: Th17 cells represent a beneficial response, while neutrophil IL-17 is associated with poor prognosis in squamous cervical cancer. Oncoimmunology.

[44] Ye J, Livergood RS, Peng G (2013). The role and regulation of human Th17 cells in tumor immunity. Am J Pathol.

[45] Murugaiyan G, Saha B (2009). Protumor vs antitumor functions of IL-17. J Immunol.

[46] Welte T, Zhang XH (2015). Interleukin-17 Could Promote Breast Cancer Progression at Several Stages of the Disease. Mediators Inflamm.

[47] Gopal R, Lin Y, Obermajer N, Slight S, Nuthalapati N, Ahmed M, Kalinski P, Khader SA (2012). IL-23-dependent IL-17 drives Th1-cell responses following Mycobacterium bovis BCG vaccination. Eur J Immunol.

[48] Khader SA, Gopal R (2010). IL-17 in protective immunity to intracellular pathogens. Virulence.

[49] Erin N, Korcum AF, Tanriover G, Kale S, Demir N, Koksoy S (2015). Activation of neuroimmune pathways increases therapeutic effects of radiotherapy on poorly differentiated breast carcinoma. Brain Behav Immun.

[50] Talebian F, Liu JQ, Liu Z, Khattabi M, He Y, Ganju R, Bai XF (2012). Melanoma cell expression of CD200 inhibits tumor formation and lung metastasis via inhibition of myeloid cell functions. PLoS One.

[51] Gorczynski RM, Chen Z, Hu J, Kai Y, Lei J (2001). Evidence of a role for CD200 in regulation of immune rejection of leukaemic tumour cells in C57BL/6 mice. Clin Exp Immunol.

[52] Simelyte E, Criado G, Essex D, Uger RA, Feldmann M, Williams RO (2008). CD200-Fc, a novel antiarthritic biologic agent that targets proinflammatory cytokine expression in the joints of mice with collagen-induced arthritis. Arthritis Rheum.

