# Supplementary material for: CD200fc enhances anti-tumoral immune response and inhibits visceral metastasis of breast carcinoma
Source: Oncotarget. 2018 Apr 10;9(27):19147–58. doi: 10.18632/oncotarget.24931 (PMC5922384; doi:10.18632/oncotarget.24931)
Supplement: Supplementary file 1 [file oncotarget-09-19147-s001.pdf]

CD200fc enhances anti-tumoral immune response and inhibits visceral metastasis of breast carcinoma

SUPPLEMENTARY MATERIALS

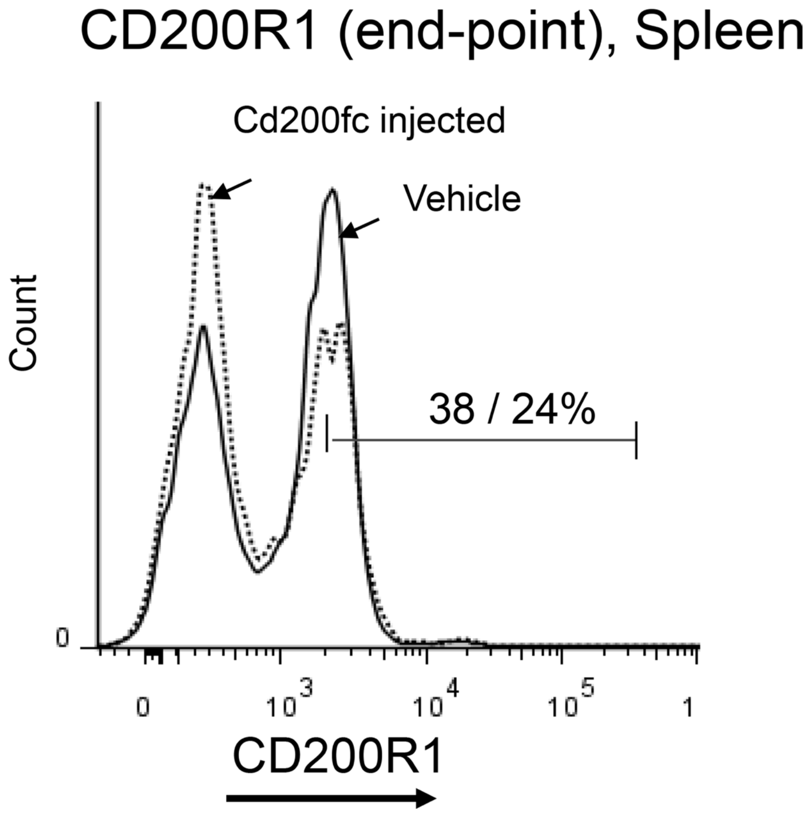

Supplementary Figure 1: Effects of CD200fc treatment on Cd200R1 expression on splenocytes.

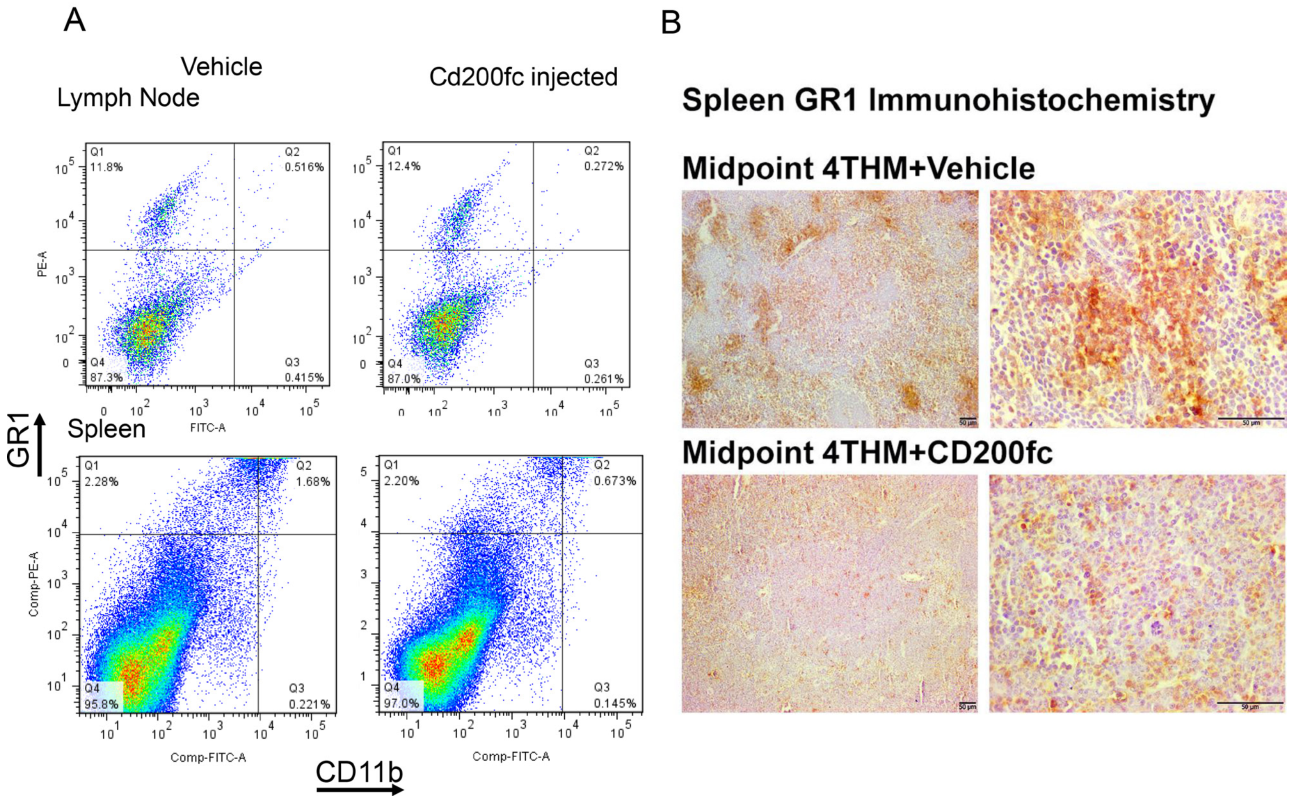

Supplementary Figure 2: (**A**) Effects of Cd200fc treatment on Gr1+Cd11b+ cells of draining lymph nodes and spleen. (**B**) Effects of CD200fc on Gr1+ cells in spleen varified by immunohistochemistry.
